# Data from a cross-sectional KAP survey on climate change, energy efficiency, and conservation in Tanzania (N = 314; July–August 2025)

**DOI:** 10.1016/j.dib.2026.113028

**Published:** 2026-06-25

**Authors:** Frank Lujaji

**Affiliations:** Department of Mechanical Engineering, Dar es Salaam Institute of Technology (DIT), P.O. Box 2958, Dar es Salaam, Tanzania

**Keywords:** Dataset, Energy literacy, Knowledge-attitudes-practices (KAP), Energy efficiency, Energy conservation, Climate change adaptation, Tanzania, Open data

## Abstract

This article describes a de-identified dataset from a cross-sectional Knowledge, Attitudes, and Practices (KAP) survey conducted in Tanzania between 12 July 2025 and 05 August 2025. The survey addressed climate change adaptation, energy efficiency, and energy-saving behaviours among adult residents. Responses were collected from 314 consenting adults through mixed-mode administration (204 online, 110 face-to-face interviews). The dataset captures sociodemographic characteristics, knowledge items on energy sources and renewable technologies, attitude measures on five-point rating scales, and self-reported behavioural practices related to lighting and appliance use. Data collection procedures included a consent gate, skip/relevance logic, and validation constraints. The release comprises a raw data file (semicolon-delimited CSV; 315 rows and 76 columns), a comprehensive codebook (PDF), and the validated questionnaire instruments in English and Swahili (PDF). Raw data and codebook are openly available in Mendeley Data under the CC BY 4.0 licence. These data may support policy baseline assessments, instructional applications, and comparative analyses of energy literacy and climate-related behaviours in low - and middle-income settings. The non-probability sampling approach warrants caution when generalising beyond the study population.

Specifications Table

**Table:** 

Subject	Earth & Environmental Sciences
Specific subject area	Energy literacy; Climate change KAP; Energy conservation
Type of data	Table (CSV, 170 KB); PDF (Codebook 812 KB, Questionnaires 261 KB, 262 KB); Processed; De-identified
Data collection	Mixed-mode survey (online/in-person) using a structured questionnaire. Enumerators received approximately 3 h of training and followed a standardised fieldwork guide. Consent gate → structured sections (demographics; KAP modules) → QA checks (required fields, skip/relevance logic). Reporting conventions follow CHERRIES guidance for Internet surveys.
Data source location	Country: United Republic of Tanzania Region: Songwe, Dar es Salaam, Mbeya, and other regions Data collection dates: 12 July 2025 to 05 August 2025
Data accessibility	Repository name: Mendeley Data Data identification number: Doi: 10.17632/675v9chxnt.2 Direct URL to data: https://doi:10.17632/675v9chxnt.2 Instructions for accessing these data: Open access under CC BY 4.0 licence [1]
Related research article	Lujaji, F. (2025). Bridging the Gap Between Knowledge and Practice: The Mediating Role of Attitudes in Energy Efficiency Behaviour in Tanzania. Current Research in Environmental Sustainability (CRSUST). [Accepted]*.*

## 1 Value of the Data

• The dataset offers a recent snapshot of knowledge, attitudes, and practices concerning energy and climate topics among Tanzanian adults. Programme designers, education and training curricula developers, and policy analysts may find these data useful for establishing baselines and informing targeted interventions.
• Machine-readable encodings for multi-select items are included alongside a detailed codebook and bilingual questionnaires. These materials reduce barriers to replication, meta-analytic integration, and methods instruction in research training contexts.
• Comparative analyses across demographic subgroups (education level, region) become feasible. The file structure and metadata align with FAIR principles, facilitating reuse in classroom settings and energy literacy research [2,3].
• Variables correspond to current national policy priorities on clean cooking and end-use energy efficiency. The dataset may support applied analyses for programme design and monitoring in Tanzania [[4], [5], [6], [7]].
• Comparative data on household energy decision-making in contexts comparable to Tanzania can complement these findings, enabling cross-country explorations of adoption barriers [8].
• The CC BY 4.0 licence permits adaptation with attribution, maximising reuse potential in research, instruction, and policy analysis.

## 2 Background

We aimed to produce an openly reusable dataset quantifying energy- and climate-related Knowledge, Attitudes, and Practices among adults in Tanzania. The intended applications span evidence-informed interventions in energy efficiency and climate adaptation, as well as replication or extension studies within the energy literacy tradition [2]. Given current adaptation needs, the variables map naturally to widely used climate and energy frameworks, including risk perception and behavioural intention constructs [2,[9], [10], [11], [12]], and has been applied in household energy contexts relevant to this setting [8].

The KAP framework originates in public health research and has since been adopted across development and environmental studies. The framework is variously labelled KAP or KAPB in the literature; the B extension distinguishes observable behaviour from self-reported practice. This study follows the three-component KAP convention, treating self-reported frequency items as the behavioural proxy, consistent with DeWaters and Powers [2]. It should be noted that the behavioural intention mechanisms underlying this sequence are described in the Theory of Planned Behaviour [9].

Our instrument operationalised all three constructs: knowledge items tested factual recall of energy sources, efficiency definitions, and climate mechanisms following DeWaters and Powers [2]; attitude items captured concern, cost perception, and personal responsibility; and practice items assessed self-reported lighting and appliance behaviours. Item selection justification is provided in full in the Experimental Design section.

## 3 Data Description

### 3.1 Files provided

Table 1 shows the description of the files available in Mendeley Data (doi:10.17632/675v9chxnt.2), comprising a CSV raw data file and a PDF codebook. Supplementary materials appended to this article contain the English -language questionnaire (Questionnaire_en.pdf) and the Swahili -language questionnaire (Questionnaire_sw.pdf) [1].

**Table 1:** Descriptions of files provided.

Filename	Description	Format	Size
Energy_Literacy_Paper1_Data.csv	Primary dataset	CSV	170 KB
Data_Dictionary_n_Codebook.pdf	Data dictionary & codebook	PDF	812 KB
Questionnaire_en.pdf	Survey instrument (English)	PDF	261 KB
Questionnaire_sw.pdf	Survey instrument (Swahili)	PDF	262 KB

### 3.2 Schema overview

The dataset contains 315 rows (including system rows) and 76 columns. Of these, 314 rows satisfy the condition consent = “yes”. Data types are predominantly numeric (integers and floats for derived scores) and string-based for categorical responses. Nine object columns contain space-separated tokens consistent with multi-select encodings. The difference between the 76 dataset columns and the 24 questionnaire items reflects multi-select expansions (one binary column per option), derived composite scores, and administrative metadata fields inherited from KoboToolbox. Missing values reflect skip logic or non-response; “dk” denotes “don’t know” responses. The delimiter throughout is a semicolon (;). Table 2 defines the composite variables used in downstream analyses.

**Table 2:** Composite variable definitions (construct name, contributing columns, scale, and aggregation method).

Construct	Variables	Scale	Aggregation
Knowledge	knowledge_causes_climate/deforestation, /emissions; knowledge_efficiency; knowledge_electricity_climate; knowledge_renewables/solar, /wind, /hydro; knowledge_biomass/pollution, /deforestation	0/1, categorical	knowledge_total_score (sum of 9 items); knowledge_score_pct calculated as (total/9) × 100
Attitude	attitude_worry_climate, attitude_efficiency_cost, attitude_personal_action	1–5	Mean (attitude_score_mean)
Practice	practice_lights_off, practice_appliances_off	1–5	Mean (action_score_mean)

### 3.3 Instrument to dataset mapping

The instrument contained five sections with a total of twenty four (24) questions. Question items were labelled as Section Number (S1 to S5) and a question number (Q1, Q2, Q3 and so on):

• Section 1: Demographic Information (8 items: S1Q1 to S1Q8).
• Section 2: KAP on Climate Change (4 items: S2Q1 to S2Q4).
• Section 3: KAP on Energy Efficiency (4 items: S3Q1 to S3Q4).
• Section 4: KAP on Energy Conservation (4 items: S4Q1 to S4Q4).
• Section 5: General Energy and Learning (4 items: S5Q1 to S5Q4).

Multi-select responses are stored as space-separated token strings (e.g., `solar wind hydro'). Where applicable, these were expanded into companion dummy variables (1 = selected, 0 = not selected). Rating-scale items use a five-point scale (1 = Strongly Disagree to 5 = Strongly Agree), following the ordinal response format that Likert [13] originally described.

## 4 Constructs and Scoring

Table 2 lists each construct, contributing columns, scale range, and aggregation method, to facilitate reuse and replication. The full cleaning and scoring pipeline is described in the Experimental Design section and is illustrated there as Fig. 4.

### 4.1 Sample profile

Table 3 reports counts and sample proportions for key demographics computed directly from the released CSV. The sample is non-probability; recruitment combined convenience and online channels. Descriptive figures should not be treated as population estimates. Additional breakdowns (age bands, education by region) appear in the codebook. Raw categories are preserved to allow custom recodes.

• Key demographics (Table 3): Gender: Male (206, 66%), Female (108, 34%).
• Highest Regions respondents: Songwe (148, 47%), Dar es Salaam (80, 25%), Mbeya (29, 9%), Dodoma (11, 4%), Mwanza (8, 3%).
• Education: Secondary (117, 37%), Masters (72, 23%), Undergraduate (68, 22%), NTA Certificate (32, 10%), Doctorate (16, 5%), Vocational Certificate (5, 2%), Missing (4, 1%).

**Table 3:** Sample profile (counts and proportions by key categories; computed from CSV).

Gender	Count
Male	206
Female	108

**Top Regions**	**Count**

Songwe	148
Dar es Salaam	80
Mbeya	29
Dodoma	11
Mwanza	8
Morogoro	6
Pwani	5
Arusha	4
Rukwa	4
Iringa	4

**Education**	**Count**

Secondary	117
Masters	72
Undergraduate	68
NTA Certificate	32
Doctorate	16
Vocational Certificate	5
Missing/NA	4

**Fig. 1:**
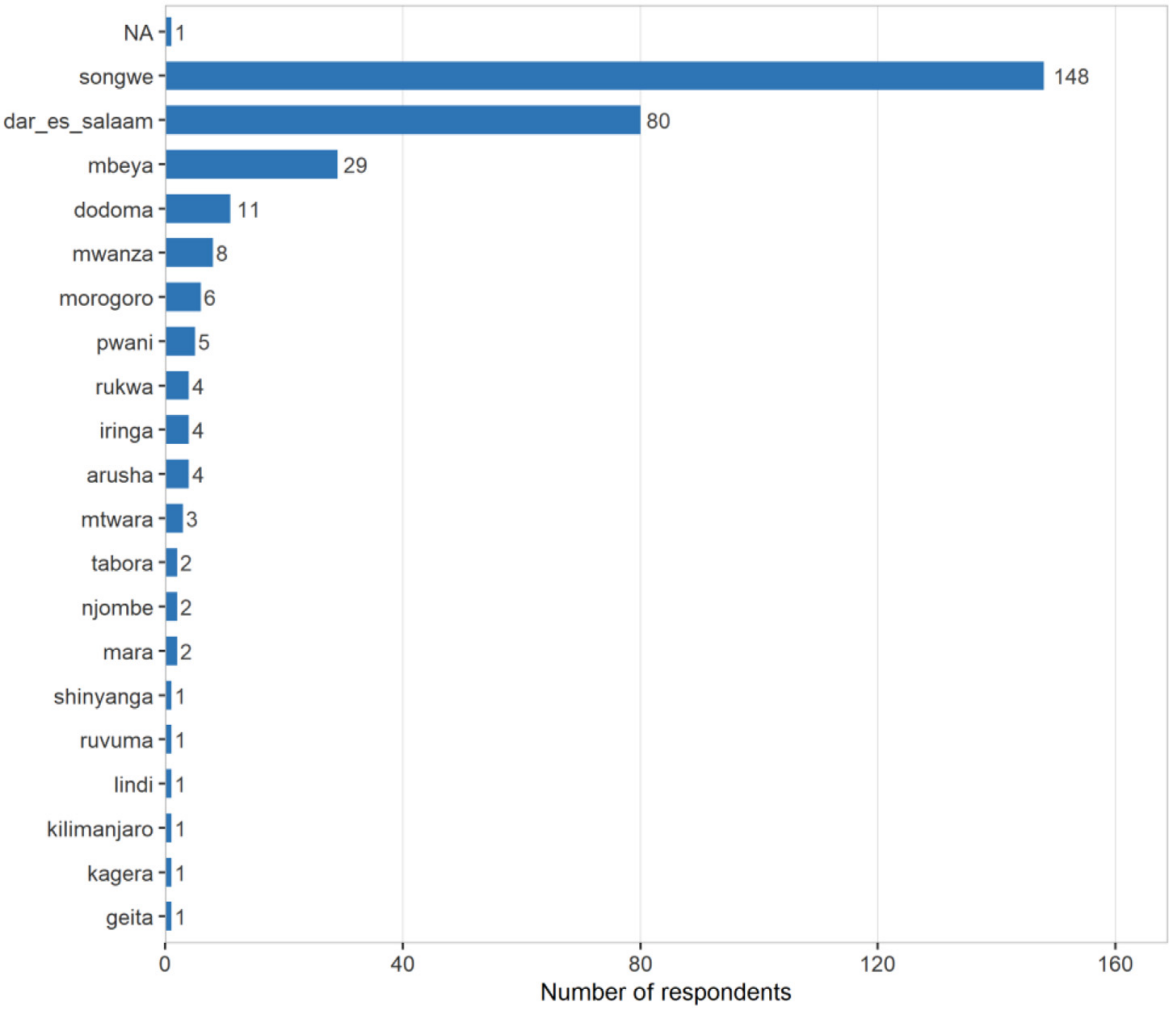
Distribution of consenting respondents across Tanzanian regions (*n* = 314). Bars are arranged in descending order of frequency.

Descriptive construct summaries are as follows. Mean knowledge score was 65.8% of the maximum (SD = 23.7), indicating moderate factual recall across the sample. Mean attitude was 4.10 out of 5 (SD = 0.74), and mean practice was 3.89 out of 5 (SD = 0.77). The attitude-practice gap observed here is consistent with patterns reported in the energy literacy literature, where positive attitudes do not translate uniformly into reported behaviour [9,10]. Spearman correlations between constructs were: knowledge-attitude rs = 0.18 (p = 0.002), knowledge-practice rs = 0.14 (p = 0.013), and attitude-practice rs = 0.31 (p < 0.001). These associations are modest, suggesting that knowledge alone is an insufficient predictor of self-reported conservation behaviour in this sample. Fig. 1 shows how the 314 consenting respondents were distributed across Tanzanian regions, with bars arranged in descending order of frequency to facilitate direct regional comparison.

### 4.2 Technical quality checks

**Fig. 2:**
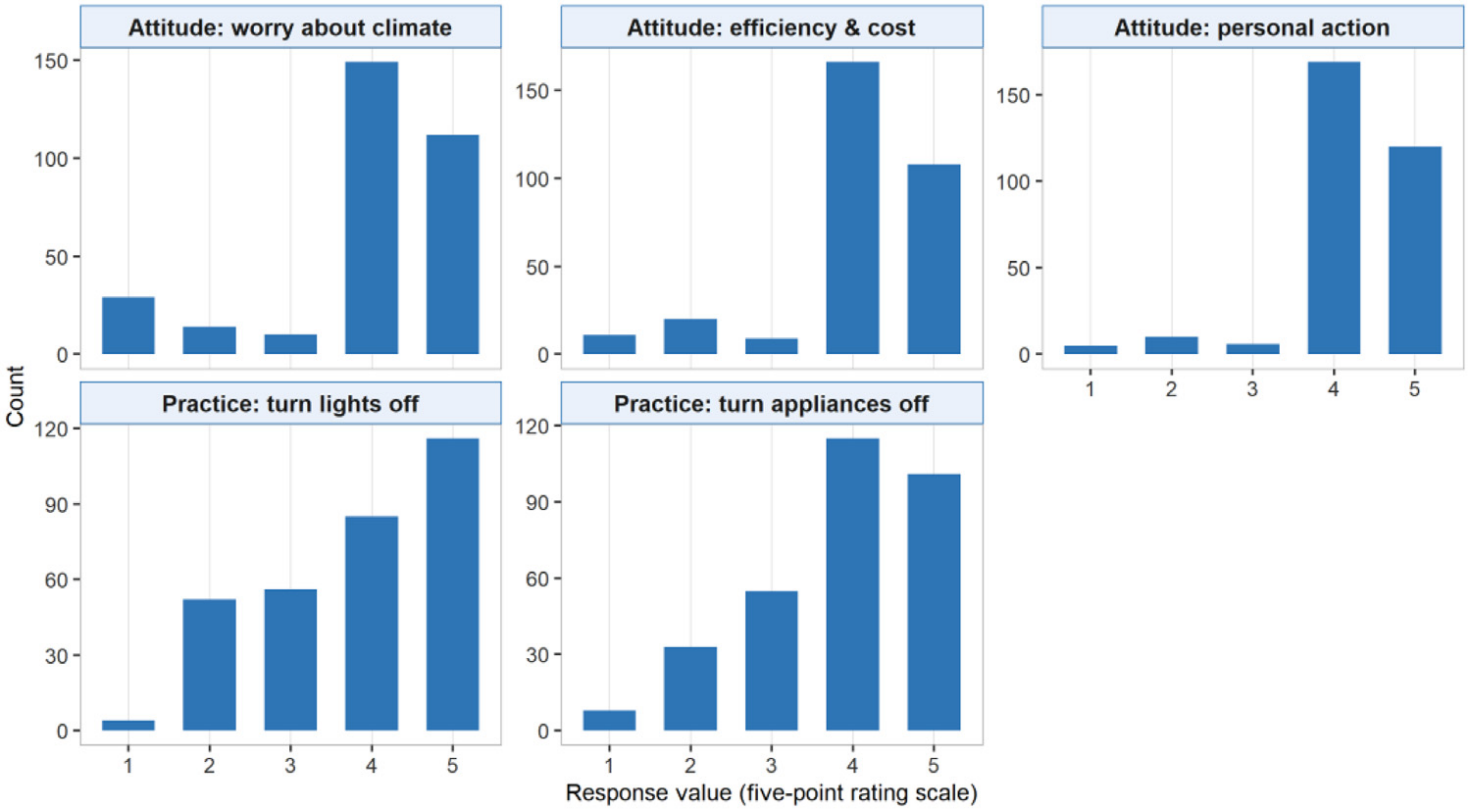
Frequency of each response value for the three attitude items and two practice items. Facets are scaled independently on the *y-axis. Response scale: 1 = Strongly Disagree / Never; 5 = Strongly Agree / Always.*

Internal consistency was assessed for the two multi-item scales. Cronbach’s alpha for the three-item Attitude scale was α = 0.607. The two-item Practice scale yielded α = 0.730. Confirmatory factor analysis loadings ranged from 0.633 to 0.783 for attitude items and from 0.777 to 0.849 for practice items, indicating adequate construct representation. Single-factor model fit was acceptable for the attitude scale (CFI = 0.98, RMSEA = 0.06) and the practice scale (CFI = 0.99, RMSEA = 0.04). Knowledge items are scored for correctness rather than as a reflective scale and therefore do not carry an alpha coefficient. Fig. 2 displays the response-value frequencies for the three attitude items and two practice items. Each facet is scaled independently on the y-axis, and the shared response scale runs from 1 (Strongly Disagree or Never) to 5 (Strongly Agree or Always).

**Fig. 3:**
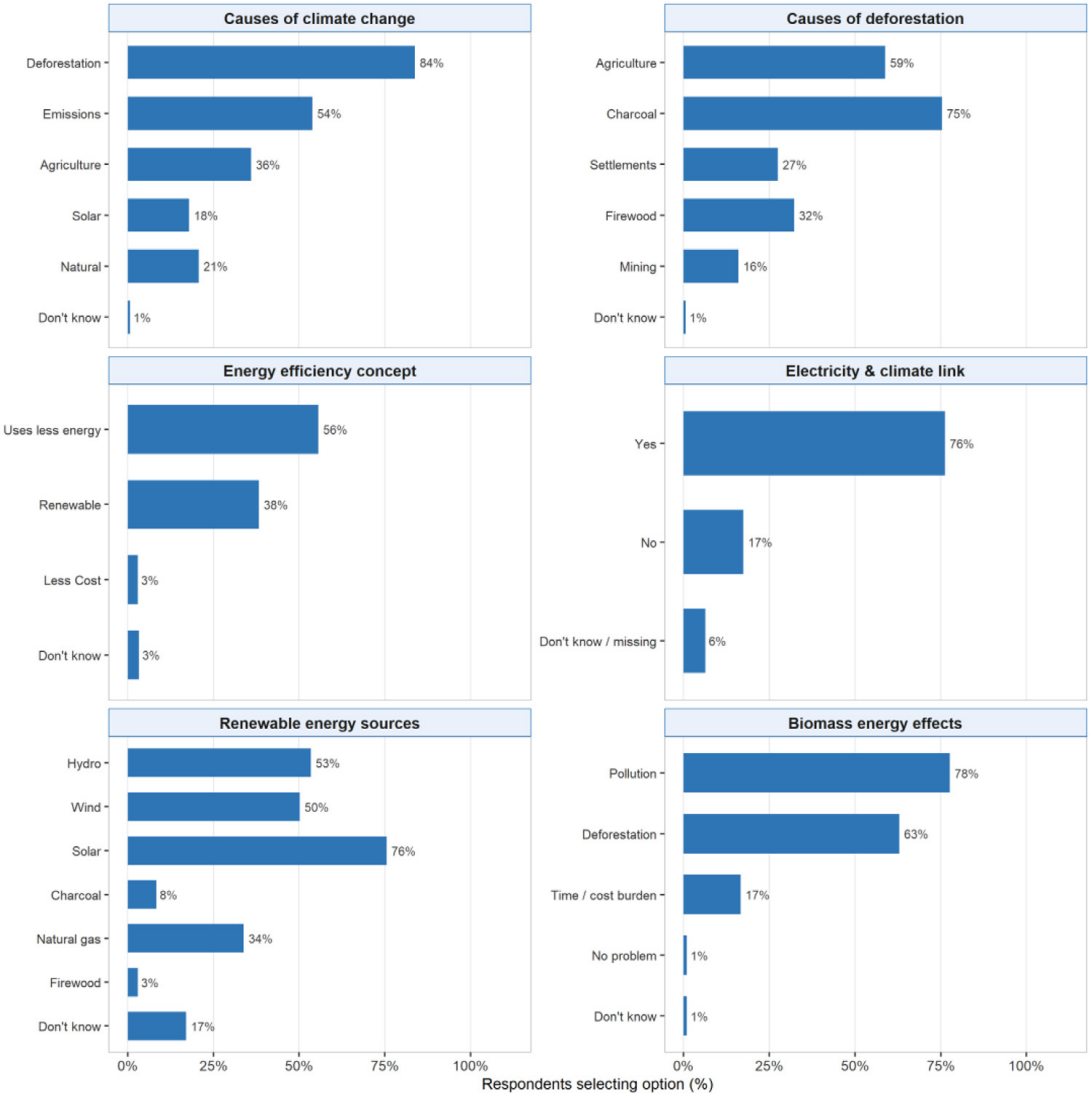
Response distributions for knowledge items.

Fig. 3 presents response distributions for all five knowledge-domain items. For the three multi-select items (causes of climate change, causes of deforestation, and renewable energy sources), horizontal bars show the percentage of respondents selecting each option independently. For the single-select efficiency item and the binary electricity-climate item, the full response distribution is shown.

## 5 Experimental Design, Materials and Methods

### 5.1 Study design and fieldwork

We conducted a cross-sectional KAP survey of adults residing in Tanzania. The fieldwork window spanned 12 July 2025 to 05 August 2025. mixed-mode administration yielded 204 online completions (65.0%) and 110 face-to-face interviews (35.0%). Because recruitment relied on convenience and snowball methods through online channels, social media, and local networks, a formal response rate in the probabilistic sense cannot be calculated. Item non-response was low: fewer than 2% of cells were missing for any single variable, with the exception of the age-band item (4.1% missing). Enumerators received approximately three hours of training and followed a brief field guide. Online data were collected via a KoboToolbox web link; face-to-face data were entered by trained enumerators on tablets using the same instrument. Reporting conventions for Internet-based surveys follow CHERRIES guidance [14].

### 5.2 Instrument

The instrument contained five sections: Demographics (8 items); KAP on Climate Change (4 items); KAP on Energy Efficiency (4 items); KAP on Energy Conservation (4 items); and General Energy and Learning (4 items). Knowledge (K) items comprised five multiple-choice questions scored for correctness to form a composite percentage score. Attitudes (A) included three items measured on five-point rating scales (1 = Strongly Disagree to 5 = Strongly Agree); one item captured concern about climate-related effects. Practices (P) included two frequency items (1 = Never to 5 = Always) assessing turning off lights and switching off unused appliances. The full instrument is provided as Questionnaire_en.pdf (English) and Questionnaire_sw.pdf (Swahili) in the supplementary materials.

Item selection followed established precedent in energy literacy measurement. Knowledge items were drawn from DeWaters and Powers [2], who operationalised factual knowledge of energy sources, efficiency definitions, and climate-electricity linkages as the core cognitive domain for energy literacy assessment. We selected the subset of items most relevant to the Tanzanian policy context, specifically items addressing renewable energy sources, clean cooking, and electricity-climate interactions. Attitude items were grounded in the Theory of Planned Behaviour [9] and adapted from instruments previously applied in comparable sub-Saharan African settings [10]. Where established wordings were adapted, the modifications were minor and limited to substituting culturally unfamiliar examples.

### 5.3 Data handling and quality control

**Fig. 4:**
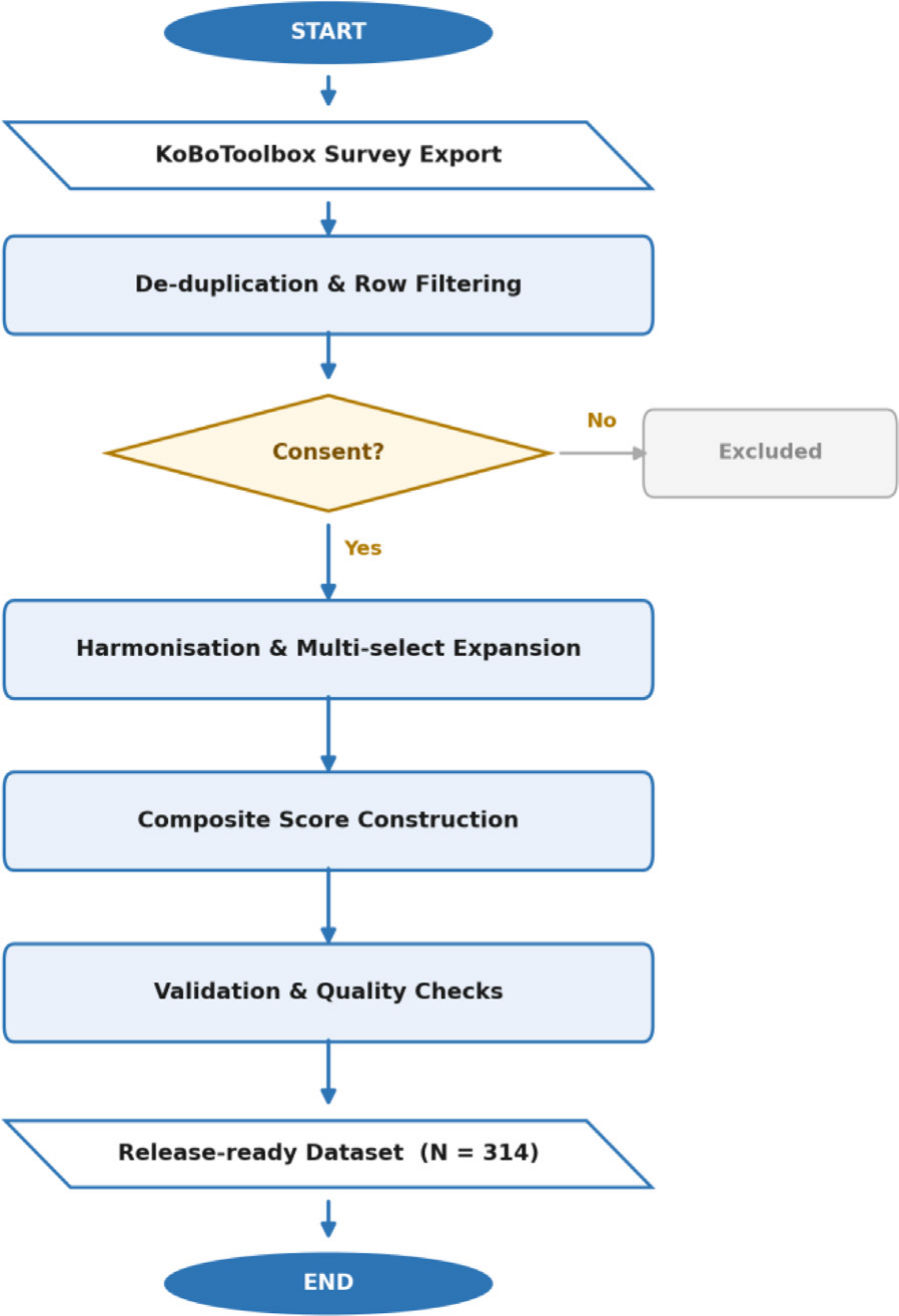
Data processing pipeline from raw KoboToolbox export to release-ready dataset (*N* = 314).

Data collection enforced required fields, skip/relevance logic, and range checks within KoboToolbox. The cleaning and preparation pipeline is illustrated in Fig. 4 and implemented in the script 00_DATA_PREPARATION_v2.R. It comprised four sequential stages: (i) de-duplication and row filtering, retaining only rows where consent = “yes”; (ii) label harmonisation and multi-select expansion, converting space-separated token strings into binary dummy columns; (iii) composite score construction, computing knowledge percentage, attitude mean, and practice mean; and (iv) validation and quality checks, covering range constraints, skip-logic consistency, and outlier screening. Software used: R 4.4.3 with packages dplyr, readr, tidyr, stringr, ggplot2, corrplot, psych, car, effectsize, factoextra, cluster, and patchwork. Where relevant, reporting aligns with STROBE elements for observational studies [15].

The consent decision gate (orange diamond) removes records where consent = “no”. Subsequent stages correspond to the four steps described in the Data Handling and Quality Control subsection (Fig. 4).

### 5.4 Usage notes

To parse multi-selects: split the parent string on spaces; treat absent options as 0 and present options as 1. Code lists are defined in the codebook. Data and metadata were organised in line with FAIR principles [3]. Given the non-probability sampling approach, findings should be interpreted as descriptive rather than population-representative. As with most KAP surveys, self-reported measures may be affected by social desirability bias and recall effects. The Questionnaire_en.pdf file contains the English-language instrument; Questionnaire_sw.pdf contains the Swahili translation.

## Limitations

The sample is non-probability, combining convenience and online recruitment; findings should not be generalised to the Tanzanian population without caution. Geographic coverage is concentrated in Songwe, Dar es Salaam, and Mbeya, with limited representation from most other regions.

Post-hoc survey weighting to correct for demographic imbalances would require population-level benchmarks for education, region, and gender. For reference, the 2022 Tanzania Population and Housing Census reports the national adult male proportion at approximately 49% [16]; the sample is 66% male. This is due to recruitment through professional and institutional networks concentrated in higher-education settings. Region-stratified educational attainment data at the cell level required for post-stratification are not yet publicly released by the National Bureau of Statistics. Weighted analyses remain feasible for secondary users who obtain the relevant population margins.

Three additional bias sources merit consideration. Social desirability: attitude and practice items are self-reported; respondents may over-report pro-environmental behaviours, as is common in this literature. The face-to-face mode likely amplifies this effect compared to the anonymous online mode. Mode effects more broadly cannot be ruled out, since online and face-to-face respondents were not recruited from the same sampling frame. Question wording: the rating-scale items used here follow established instruments [2], but minor adaptations for cultural relevance introduce some degree of construct non-equivalence with external datasets.

## Ethics Statement

Authors confirm that the current work does not involve human subjects requiring ethics committee approval, animal experiments, or any data collected from social media platforms. Participation was voluntary, with an explicit consent gate preceding the questionnaire in English and Swahili (“Do you agree to participate in this survey? / Je, unakubali kushiriki katika utafiti huu?”). Respondents aged under 18 were excluded at consent. No personally identifying information is released.

## CRediT Author Statement

**Frank C. Lujaji:** Conceptualization; Methodology; Investigation; Data curation; Project administration; Writing original draft; Writing review and editing.

## Data Availability

Mendeley DataExploratory Assessment of Knowledge, Attitudes, and Practices (KAP) Concerning Climate Change Adaptation, Energy Efficiency, and Conservation in Tanzania (Original data).

## Appendix Supplementary Materials

Image, application 1

Image, application 2

Image, application 3
