# Supplementary material for: Data from a cross-sectional KAP survey on climate change, energy efficiency, and conservation in Tanzania (N = 314; July–August 2025)
Source: Data Brief. 2026 Jun 25;67:113028. doi: 10.1016/j.dib.2026.113028 (PMC13342880; doi:10.1016/j.dib.2026.113028)
Supplement: Supplementary file 2 [file mmc2.pdf]

Energy Questionnaire (Dodoso kuhusu Maswala ya Nishati) - Public

INTRODUCTION:

*Hello! My name is **Frank Lujaji**, researcher from the Dar es Salaam Institute of Technology (DIT). We are conducting a nationwide survey to better understand what people in Tanzania know, think, and do about climate change and energy use. Your participation is completely voluntary. The survey will take about 15-20 minutes. There are no right or wrong answers; we are only interested in your personal experiences and opinions. The information you provide will be kept completely anonymous and confidential. Your name will not be recorded, and your responses will be combined with those from many other people for statistical analysis only. The findings will help inform government policies to improve energy access and protect our environment. You can choose to stop the interview at any time without any consequences. Do you have any questions for me?*

DO YOU AGREE TO PARTICIPATE IN THIS SURVEY? *

- (●) Yes, I agree to participate
- ( ) No, I do not wish to participate

Section 1: Demographic Information

S1Q1: PLEASE SELECT YOUR GENDER.

- Male
- Female
- Prefer not to answer

S1Q2: WHAT IS YOUR YEAR OF BIRTH?

Please enter a valid four-digit year, for example 1985.

S1Q3: WHERE DO YOU LIVE AT THE MOMENT? *

- Urban
- Rural

S1Q4: PLEASE SELECT YOUR REGION *

Please start typing, a List will appear for selection

S1Q5: PLEASE SELECT YOUR DISTRICT *

S1Q6: WHAT IS YOUR EDUCATION STATUS?

Please select only one that apply.

- Currently studying
- Completed Studies
- No formal education

S1Q6A: PLEASE SELECT THE EDUCATION LEVEL

Please select only one that apply.

- Primary School
- Secondary School
- Vocation/Certificate (NVA 1 to 3)
- Certificate / Technitian / Diploma Level (NTA4 to NTA 6)
- Undergraduate Level
- Masters Level
- Doctorate and PhD Level

S1Q6B: WHAT IS THE CURRENT EDUCATION LEVEL YOU ARE STUDYING?

Please select only one that apply.

S1Q6C: WHAT IS THE HIGHEST EDUCATION LEVEL YOU'VE COMPLETED?

Please select only one that apply.

S1Q7: WHAT IS YOUR MAIN SOURCE OF LIVELIHOOD?

- ○ Farming / Agriculture
- ○ Livestock keeping
- ○ Salaried employee (government or private)
- ○ Self-employed / Business owner / Farmer
- ○ Student
- ○ Unemployed
- ○ Other

PLEASE SPECIFY OTHER OCCUPATION.

S1Q8: GPS LOCATION (OPTIONAL)

latitude (x.y °)

longitude (x.y °)

altitude (m)

accuracy (m)

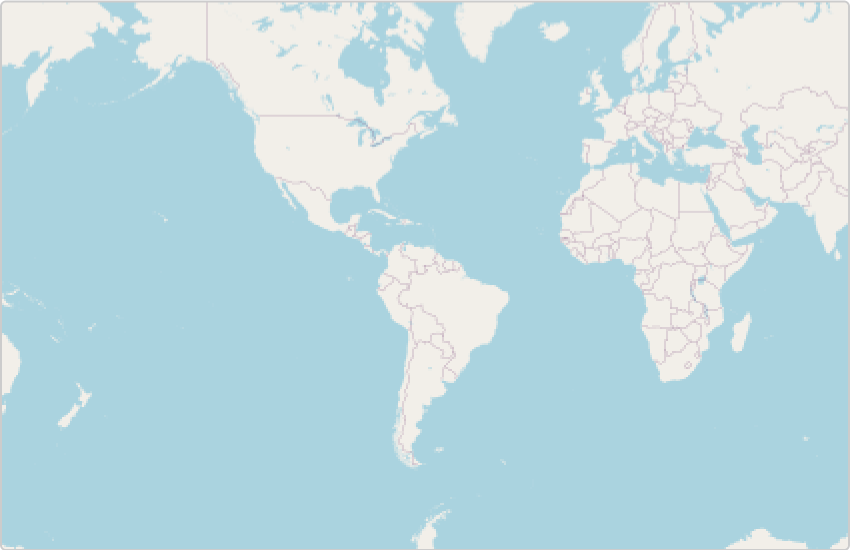

Section 2: KAP on Climate Change

S2Q1: WHICH OF THE FOLLOWING DO YOU THINK ARE THE MAIN CAUSES OF CLIMATE CHANGE?

Please select all that apply.

- [ ] Cutting down forests (deforestation)
- [ ] Smoke from factories and cars (greenhouse gas emissions)
- [ ] Use of chemical fertilizers in agriculture
- [ ] Changes in the sun's energy
- [ ] Natural cycles of the Earth
- [ ] Don't Know

S2Q2: WHAT ARE THE MAIN CAUSES OF DEFORESTATION IN TANZANIA?

Please select up to THREE.

- [ ] Clearing land for agriculture
- [ ] Production of charcoal
- [ ] Collection of firewood
- [ ] Expansion of settlements and infrastructure
- [ ] Mining activities
- [ ] Don't Know

S2Q3: "I AM WORRIED ABOUT THE NEGATIVE EFFECTS OF CLIMATE CHANGE (LIKE DROUGHTS AND FLOODS) ON MY FAMILY AND COMMUNITY."

- ○ Strongly Disagree
- ○ Disagree
- ○ Neutral
- ○ Agree
- ○ Strongly Agree

S2Q4: IN THE LAST YEAR, HAS YOUR HOUSEHOLD TAKEN ANY OF THE FOLLOWING ACTIONS TO ADAPT TO CLIMATE CHANGES?

Please select all that apply.

- [ ] Planted trees
- [ ] Used drought-resistant crop varieties
- [ ] Practiced water harvesting (e.g., collecting rainwater)
- [ ] Changed farming practices
- [ ] None of the above
- [ ] Don't Know

Section 3: KAP on Energy Efficiency

S3Q1: IF A MACHINE IS DESCRIBED AS "ENERGY EFFICIENT," WHAT DOES THAT MEAN?

- ◯ It uses less energy to do the same amount of work.
- ◯ It uses a renewable source of energy.
- ◯ It costs less money to buy.
- ◯ Don't Know

S3Q2: "IT IS IMPORTANT FOR MY HOUSEHOLD TO USE ENERGY-EFFICIENT APPLIANCES (LIKE IMPROVED COOKSTOVES OR LED BULBS), EVEN IF THEY COST MORE TO BUY."

- ◯ Strongly Disagree
- ◯ Disagree
- ◯ Neutral
- ◯ Agree
- ◯ Strongly Agree

S3Q3: WHAT TYPE OF LIGHTING DO YOU PRIMARILY USE IN YOUR HOME?

- ◯ Energy-saving bulbs / Lights - types of Light Emitting Diode (LED) or Compact Fluorescent Lamp (CFL)
- ◯ Incandescent (filament) bulbs which produce light and heat
- ◯ Kerosene lamp
- ◯ Candles
- ◯ Other

PLEASE SPECIFY OTHER LIGHTING TYPE.

....

S3Q4: DOES YOUR HOUSEHOLD USE AN IMPROVED OR ENERGY-SAVING COOKSTOVE?

- ◯ Yes
- ◯ No
- ◯ Don't know / Not applicable

Section 4: KAP on Energy Conservation

S4Q1: "SAVING ELECTRICITY CAN HELP REDUCE THE IMPACTS OF CLIMATE CHANGE."

- TRUE
- FALSE
- Don't Know

S4Q2: "MY PERSONAL ACTIONS CAN MAKE A REAL DIFFERENCE IN SAVING ENERGY FOR THE COUNTRY."

- Strongly Disagree
- Disagree
- Neutral
- Agree
- Strongly Agree

S4Q3: HOW OFTEN DO YOU TURN OFF LIGHTS WHEN YOU ARE THE LAST PERSON TO LEAVE A ROOM?

- Never
- Rarely
- Sometimes
- Often
- Always

S4Q4: HOW OFTEN DO YOU TURN OFF APPLIANCES LIKE TVS, RADIOS, OR PHONE CHARGERS AT THE WALL WHEN THEY ARE NOT IN USE?

- Never
- Rarely
- Sometimes
- Often
- Always

Section 5: General Energy and Learning

S5Q1: WHAT ARE THE PRIMARY SOURCES OF ENERGY YOUR HOUSEHOLD USES FOR COOKING?

Please select all that apply.

- [ ] Charcoal
- [ ] Firewood
- [ ] LPG (Gas)
- [ ] Electricity
- [ ] Kerosene
- [ ] Crop residues / Animal waste
- [ ] Other

PLEASE SPECIFY OTHER COOKING ENERGY SOURCE.

S5Q2: WHICH OF THESE ARE RENEWABLE ENERGY SOURCES?

Please select all that are naturally replenished. Remember that hydropower comes from dams/rivers which could be the source of Electricity.

- [ ] Solar
- [ ] Wind
- [ ] Hydropower (from dams)
- [ ] Charcoal
- [ ] Firewood
- [ ] Natural Gas
- [ ] Don't Know

S5Q3: WHICH OF THESE PROBLEMS CAN BE CAUSED BY THE WIDESPREAD USE OF CHARCOAL AND FIREWOOD FOR COOKING?

Please select all that apply.

- [ ] Indoor air pollution and respiratory problems
- [ ] Deforestation
- [ ] It takes a lot of time to collect or is expensive
- [ ] It does not cause any problems
- [ ] Don't Know

S5Q4: WHERE DO YOU LEARN THE MOST ABOUT ENERGY-RELATED ISSUES?

- ○ Radio / Television / Newspapers
- ○ Internet / Social Media
- ○ School / Educational Institutions
- ○ Family / Friends / Community members
- ○ Government announcements
- ○ I do not receive information about energy issues

THANK YOU VERY MUCH FOR YOUR TIME AND VALUABLE CONTRIBUTION.

Thank you
