# Supplementary material for: Data from a cross-sectional KAP survey on climate change, energy efficiency, and conservation in Tanzania (N = 314; July–August 2025)
Source: Data Brief. 2026 Jun 25;67:113028. doi: 10.1016/j.dib.2026.113028 (PMC13342880; doi:10.1016/j.dib.2026.113028)
Supplement: Supplementary file 3 [file mmc3.pdf]

Energy Questionnaire (Dodoso kuhusu Maswala ya Nishati) - Public

UTANGULIZI:

*Habari! Jina langu ni **Frank Lujaji**, mtafiti kutoka taasisi ya Teknolojia ya Dar es Salaam. Tunafanya utafiti wa kitaifa ili kuelewa jinsi ambavyo watu mbalimbali nchini Tanzania wanafahamu, au kufikiri, na Matendo / Mwenendo wao kuhusu mabadiliko ya tabianchi na matumizi ya nishati. Ushiriki wako ni wa hiari kabisa. Utafiti huu unafanyika kupitia maswali haya, muda wa kujibu utakuwa takribani dakika 15-20. Hakuna majibu sahihi au yasiyo sahihi; tunaomba tu kufahamu uzoefu na maoni yako binafsi. Majibu na taarifa utakazotoa zitahifadhiwa kwa usiri na bila jina lako kujulikana. Jina lako halitaandikwa, na majibu yako yatajumuishwa na ya watu wengine wengi kwa ajili ya uchambuzi wa takwimu pekee. Matokeo ya utafiti yatasaidia kuarifu sera mbali mbali za Serikali ili kuboresha upatikanaji wa nishati na kulinda mazingira yetu. Unaweza kuchagua kusitisha mahojiano haya wakati wowote. Je, una maswali yoyote kwangu?*

JE, UNAKUBALI KUSHIRIKI KATIKA UTAFITI HUU? *

- (●) Ndiyo, nakubali kushiriki
- ( ) Hapana, sitaki kushiriki

Sehemu ya 1: Taarifa za Kidemografia

S1Q1: TAFADHALI CHAGUA JINSIA YAKO.

- ◯ Mwanaume
- ◯ Mwanamke
- ◯ Napenda kuacha kujibu

S1Q2: UMEZALIWA MWAKA GANI?

Tafadhali jaza tarakimu nne, kwa mfano 1985

S1Q3: JE, MAHALA UNAPOISHI SASA NI? *

- ◯ Mjini
- ◯ Kijijini

TAFADHALI CHAGUA MKOA UNAPOISHI SASA. *

Tafadhali anza kuandika na uchague kutoka kwenye orodha

TAFADHALI CHAGUA WILAYA UNAPOISHI SASA. *

S1Q6: HALI YAKO YA ELIMU NI IPI?

Chagua moja tu inayohusika.

- ◯ Ninaendelea na Masomo kwa sasa
- ◯ Nimeshamaliza masomo / Nimehitimu
- ◯ Sijasoma elimu ya mfumo rasmi

S1Q6A: TAFADHALI CHAGUA NGAZI YA ELIMU

Chagua moja tu inayohusika.

- ◯ Shule ya Msingi
- ◯ Shule ya Sekondari
- ◯ Ufundi Stadi / Cheti (NVA 1 to 3)
- ◯ Ngazi ya Cheti / Usanifu / Stashahada (NTA 4 to 6)
- ◯ Shahada ya Kwanza
- ◯ Shahada ya Uzamili
- ◯ Shahada ya Uzamivu / PhD

S1Q6B: UNASOMA DARASA / MWAKA WA NGAPI?

Chagua moja tu inayohusika.

S1Q6C: UMEMALIZA KIWANGO GANI CHA JUU CHA ELIMU?

Chagua moja tu inayohusika.

S1Q7: KAZI YAKO KUBWA INAYOKUPATIA KIPATO NI IPI?

- ○ Kilimo
- ○ Ufugaji
- ○ Mwajiriwa (serikalini au binafsi)
- ○ Nimejiajiri / Mfanyabiashara / Mkulima
- ○ Mwanafunzi
- ○ Sina ajira
- ○ Nyingine

TAFADHALI TAJA KAZI NYINGINE ZINAZOKUINGIZIA KIPATO UNAZOZIFANYA.

....

S1Q8: ENEO LA GPS (SI LAZIMA / KWA HIARI)

latitude (x.y °)

....

longitude (x.y °)

....

altitude (m)

....

accuracy (m)

....

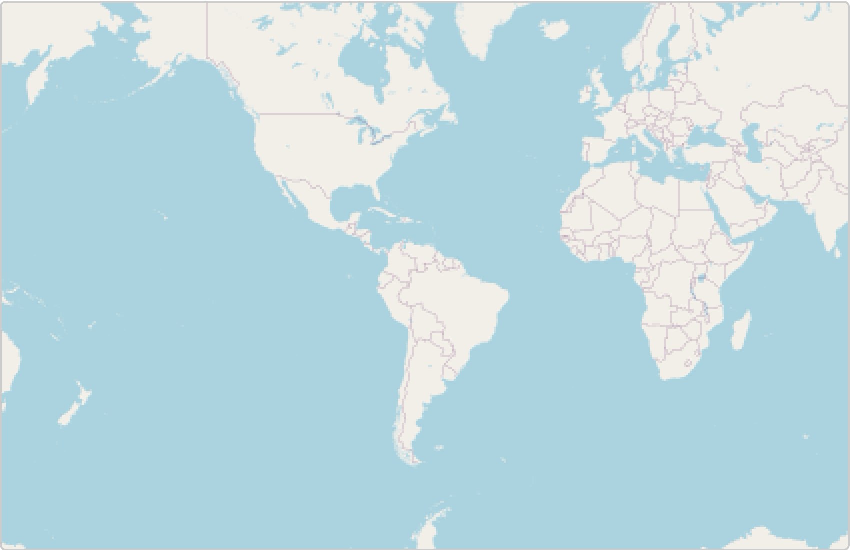

....

Sehemu ya 2: Ufahamu, Mitazamo na Matendo/Mwenendo (KAP) kuhusu Mabadiliko ya Tabianchi

S2Q1: KATI YA VIFUATAVYO, UNADHANI NI VIPI VYANZO VIKUU VYA MABADILIKO YA TABIANCHI?

Chagua zote zinazohusika.

- [ ] Ukataji miti (uharibifu wa misitu)
- [ ] Moshi kutoka viwandani na magari
- [ ] Matumizi ya mbolea za kemikali mashambani
- [ ] Mabadiliko katika nishati ya jua
- [ ] Mizunguko ya asili / Majira ya Dunia
- [ ] Sijui

S2Q2: NI VIPI VYANZO VIKUU VYA UKATAJI MITI NCHINI TANZANIA?

Chagua hadi VITATU.

- [ ] Kuandaa mashamba kwa ajili ya kilimo
- [ ] Uzalishaji wa mkaa
- [ ] Ukusanyaji wa kuni
- [ ] Upanuzi wa makazi na miundombinu
- [ ] Shughuli za uchimbaji madini
- [ ] Sijui

S2Q3: "NINA WASIWASI KUHUSU MADHARA YA MABADILIKO YA TABIANCHI (KAMA UKAME NA MAFURIKO) KWA FAMILIA NA JAMII INAYONIZUNGUKA."

- ◯ Sikubaliani kabisa
- ◯ Sikubaliani
- ◯ Sipo upande wowote
- ◯ Nakubali
- ◯ Nakubali kabisa

S2Q4: KATIKA MWAKA ULIOPITA, JE, NYUMBANI KWENU UMECHUKUA HATUA ZOZOTE KATI YA HIZI KUKABILIANA NA MABADILIKO YA TABIANCHI?

Chagua zote zinazohusika.

- ☐ Nimepanda miti
- ☐ Nimetumia mbegu/mimea inazostahimili ukame
- ☐ Ninafanya uvunaji wa maji (kwa mfano kukusanya / kuvuna maji ya mvua)
- ☐ Nimebadilisha mbinu za kilimo
- ☐ Hakuna kati ya hizi
- ☐ Sijui

Sehemu ya 3: KAP kuhusu Ufanisi wa Nishati

S3Q1: IKIWA MASHINE INAELEZWA KUWA NA "UFANISI WA NISHATI," INAMAANISHA NINI?

- Inatumia nishati kidogo kufanya kazi ileile.
- Inatumia chanzo cha nishati jadidifu, yaani "renewable source of energy"
- Ni gharama nafuu kununua.
- Sijui

S3Q2: "NI MUHIMU KWA NYUMBANI KUTUMIA VIFAA VYENYE UFANISI WA NISHATI (KAMA MAJIKO SANIFU AU TAA ZA LED), HATA KAMA GHARAMA ZA KUNUNUA VIFAA HIVI NI GHALI ZAIDI."

- Sikubaliani kabisa
- Sikubaliani
- Sipo upande wowote
- Nakubali
- Nakubali kabisa

S3Q3: UNATUMIA ZAIDI AINA GANI YA TAA NYUMBANI KWENU?

- Taa za Umeme zinazookoa nishati, aina ya - "Light Emitting Diode (LED)" au "Compact Fluorescent Lamp (CFL)"
- Taa / Balbu zenye waya maalumu ndani unaowaka kuleta mwanga na joto.
- Taa ya mafuta ya taa
- Mishumaa
- Nyingine

TAFADHALI TAJA AINA NYINGINE YA TAA.

S3Q4: JE, NYUMBANI KWENU UNATUMIA JIKO SANIFU (JIKO BORA) AU JIKO LINALOOKOA NISHATI?

- Ndiyo
- Hapana
- Sijui / Haihusiki

Sehemu ya 4: KAP kuhusu Uhifadhi wa Nishati

S4Q1: "KUOKOA UMEME KUNAWEZA KUSAIDIA KUPUNGUZA MADHARA YA MABADILIKO YA TABIANCHI."

- Kweli
- Si kweli
- Sijui

S4Q2: "UTASHI / TABIA YANGU BINAFSI INAWEZA KULETA MABADILIKO HALISI KATIKA KUOKOA MATUMIZI MAKUBWA YA NISHATI KUSAIDIA TAIFA."

- Sikubaliani kabisa
- Sikubaliani
- Sipo upande wowote
- Nakubali
- Nakubali kabisa

S4Q3: NI MARA NGAPI UNAZIMA TAA UNAPOKUWA MTU WA MWISHO KUTOKA CHUMBA AMA ENEO LENYE TAA?

- Kamwe
- Mara chache
- Mara kwa mara
- Mara nyingi
- Kila mara

S4Q4: NI MARA NGAPI UNAZIMA VIFAA KAMA TV, REDIO, AU CHAJA ZA SIMU UKUTANI WAKATI HAVITUMIKI?

- Kamwe
- Mara chache
- Mara kwa mara
- Mara nyingi
- Kila mara

Sehemu ya 5: Nishati kwa Ujumla na Taarifa/Elimu ya Maswala ya Nishati

S5Q1: NI VYANZO VIPI VYA NISHATI AMBAVYO MNATUMIA NYUMBANI KWA KUPIKIA?

Chagua zote zinazohusika.

- [ ] Mkaa
- [ ] Kuni
- [ ] Gesi (LPG)
- [ ] Umeme
- [ ] Mafuta ya taa
- [ ] Mabaki ya mazao / Samadi
- [ ] Nyingine

TAFADHALI TAJA CHANZO KINGINE CHA NISHATI YA KUPIKIA.

....

S5Q2: KATI YA HIVI, NI VIPI VYANZO VYA NISHATI JADIDIFU?

Chagua zote zinazoweza kurejea tena kwenye hali yake ya mwanzo kwa njia ya asili. Kumbuka kwamba Nishati ya Maji (Maporomoko ya Mito) inaweza kuzalisha umeme.

- [ ] Nishati ya Jua (Sola)
- [ ] Nishati ya Upepo
- [ ] Nishati ya Maji (kutoka kwenye maporomoko ya maji mitoni)
- [ ] Mkaa
- [ ] Kuni
- [ ] Gesi Asilia
- [ ] Sijui

S5Q3: NI MATATIZO YAPI KATI YA HAYA YANAWEZA KUSABABISHWA NA MATUMIZI MAKUBWA YA MKAA NA KUNI KWA KUPIKIA?

Chagua zote zinazohusika.

- [ ] Uchafuzi wa hewa ndani ya nyumba na magonjwa ya kupumua
- [ ] Ukataji miti
- [ ] Inachukua muda mwingi kukusanya au ni ghali
- [ ] Haisababishi matatizo yoyote
- [ ] Sijui

S5Q4: UNAJIFUNZA / KUPATA WAPI ELIMU ZAIDI KUHUSU MASUALA YA NISHATI?

- ○ Redio / Televisheni / Magazeti
- ○ Intaneti / Mitandao ya kijamii
- ○ Shuleni / Taasisi za kielimu
- ○ Familia / Marafiki / Wajumbe wa jamii
- ○ Matangazo ya Serikali
- ○ Sipati taarifa kuhusu masuala ya nishati

ASANTE SANA KWA MUDA WAKO NA MCHANGO WAKO MUHIMU.

Ahsante
